# Correlation Between Irisin and Cognitive Functions in Alzheimer Dementia

**DOI:** 10.1002/acn3.70117

**Published:** 2025-06-25

**Authors:** Patrizia Pignataro, Manuela Dicarlo, Chiara Zecca, Daniele Urso, Maria Teresa Dell'Abate, Davide Vilella, Francesco Borlizzi, Roberta Zerlotin, Angela Oranger, Graziana Colaianni, Silvia Colucci, Maria Grano, Giancarlo Logroscino

**Affiliations:** ^1^ Department of Translational Biomedicine and Neuroscience (DiBraiN) University of Bari “A. Moro” Bari Italy; ^2^ Department of Precision and Regenerative Medicine and Ionian Area (DiMePRe‐J) University of Bari “A. Moro” Bari Italy; ^3^ Center for Neurodegenerative Diseases and the Aging Brain University of Bari “A. Moro” at “pia Fondazione Card G. Panico” Hospital Bari Italy

**Keywords:** Alzheimer's disease, dementia, irisin, mild cognitive impairment, neurodegeneration

## Abstract

**Objective:**

The myokine irisin, a recent positive mediator of exercise in the brain, shows neuroprotective functions against Alzheimer's disease (AD). The association between irisin and cognition has never been explored in a biologically defined cohort of patients. Thus, in the present study, we investigated the association of irisin with multidomain cognition in patients showing dementia‐related symptomatology.

**Methods:**

Cerebrospinal fluid (CSF) and serum irisin levels were evaluated using enzyme‐linked immunoassays in a cohort of subjects with a confirmed biomarker evidence of AD, including AD (*n* = 82), mild cognitive impairment (MCI, *n* = 44), and subjective memory complaint (SMC, *n* = 20) patients. The results of this analysis were correlated with global cognitive efficiency assessed by Mini‐Mental State Examination, and multidomain cognition evaluated by a battery of psychometric tests.

**Results:**

Decreased CSF and serum irisin levels were observed in AD and MCI patients compared to SMC. A significant correlation has been found between irisin in the CSF and global cognitive efficiency, as well as with specific cognitive domains such as memory, executive functions, attention, visuospatial abilities, and language. For serum irisin, the correlation analysis evidenced similar results to those observed for the CSF.

**Interpretation:**

Our results highlight the key involvement of irisin in multidomain cognition, indicating its potential role as a cognitive biomarker of AD progression.

## 1 Introduction

Over the past century, increased life expectancy has significantly raised the number of people over 65 years of age worldwide, and this number is predicted to continue rising in the coming decades. However, as a downside, a longer lifespan and population growth led to an increased occurrence of age‐related diseases, including the neurodegenerative disorders characterized by the progressive decline of cognitive functions [1, 2]. Among them, Alzheimer's disease (AD) is the most common as it accounts for 80% of dementia in elderly subjects and still has no effective treatments to reverse and/or slow its progression [3]. The high costs and poor availability of new drugs, such as antiamyloid antibodies, have prompted research into other approaches including preventive strategies and lifestyle interventions to stave off AD [4]. Regular physical exercise (PE) has emerged as one of the lifestyle factors that has the potential to improve cognitive functions in normal subjects with high risk of AD and in patients with early stage of AD [5, 6, 7]. Several pieces of evidence showed the role of fibronectin type III domain‐containing protein 5 (FNDC5), a muscle glycosylated type 1 membrane protein, and its circulating product irisin, in the improvement of cognitive functions induced by PE [8]. Indeed, first, Wrann et al. demonstrated the beneficial effect of the FNDC5/irisin pathway on brain functions, showing the increased expression of FNDC5 and other neuroprotective genes, such as the neurotrophin brain‐derived neurotrophic factor (BDNF), in the hippocampus of mice submitted to endurance exercise [9]. Very recently, it has been reported that irisin is the active moiety of the cognitive benefits of PE as the deletion of the Fndc5/irisin gene impaired cognitive functions in exercise and aging [10]. Conversely, irisin restored the cognitive functions in two different AD mouse models if directly delivered into the dentate gyrus or peripherally expressed by adenoviral vectors in the liver [10]. In parallel studies on cohorts of AD patients, we and others have evidenced the association between irisin levels in the cerebrospinal fluid (CSF) and AD biomarkers, such as amyloid‐β (Aβ) and tau proteins [11, 12]. Only a few studies focused on the relationship between irisin and cognition in AD patients, evidencing the correlation of CSF or plasma irisin with global cognition [13, 14]. However, these reports did not examine individual cognitive domains in a cohort of biologically characterized patients [13, 14].

Therefore, in this study, to elucidate the cognitive profile linked with irisin, we analyzed the relationship between CSF and serum irisin levels and the unexplored individual cognitive domains, including memory, executive function, attention, visuospatial abilities, and language. Thus, to clarify the mechanistic link between irisin and AD, we studied a biologically defined cohort of 146 patients according to the amyloid/tau/neurodegeneration (ATN) scheme of the National Institute on Aging–Alzheimer's Association (NIA‐AA) [15].

## 2 Methods

### 2.1 Participants

This study was conducted on a cohort of 146 participants with diagnosis of AD (AD dementia, *n* = 82), mild cognitive impairment (MCI, *n* = 44), and subjective memory complaint (SMC, *n* = 20). This cohort was used in a previous work that we conducted showing the correlation between CSF irisin levels and AD biomarkers [12]. All patients were enrolled at the Center for Neurodegenerative Diseases and the Aging Brain of the University of Study of Bari “Aldo Moro” at Pia Fondazione “Card. Panico” Hospital (Tricase) and were submitted to a multidisciplinary examination including neurological and neuropsychological tests, nutritional assessment, 3 T magnetic resonance imaging scan, routine laboratory analysis, and lumbar puncture for CSF biomarkers analysis. For AD dementia diagnosis, we used the standard diagnostic criteria for dementia (Diagnostic and Statistical Manual of Mental Disorders, 5th edition [DSM‐V]) [16] and the NIA‐AA criteria [15, 17]. For MCI, the prodromal stage of AD, the diagnosis was performed according to the DSM‐V criteria: (a) change in cognitive abilities noted by patient, informant or clinician, (b) cognitive impairment in one or more domains (e.g., memory, language, executive functions) confirmed by neuropsychological tests, (c) preserved daily activities, and (d) absence of dementia. We also considered the presence of biomarkers that reflect AD pathology, such as the Aβ and tau. All patients with AD and MCI had evidence of Alzheimer pathology [15].

For SMC, the group including cognitively normal subjects, with no significant impairment in cognitive functions and in their daily living activities, the diagnosis was based on the following criteria: (a) persistent memory and cognitive abilities decline self‐reported compared to a previously normal state and not induced by an acute event; and (b) normal performance confirmed by standardized neuropsychological tests used to classify MCI or prodromal AD [18]. To rate dementia and cognitive impairment severity, the Clinical Dementia Rating (CDR) scale and CDR scale Sum of Boxes (CDR‐SOB) were used, respectively.

This study was approved by the local ethical committee (ASL Lecce verbale No. 6, May 25, 2017), according to the Declaration of Helsinki [19], and all patients provided their written informed consent. The participants of this study were only enrolled based on their clinical diagnosis, without age, sex, and race discrimination. In line with this aspect, the authors recruited patients ensuring sex and gender balance to avoid any form of discrimination.

### 2.2 Cognitive Assessment

Each patient underwent an extensive neuropsychological evaluation. The protocol included 18 psychometric tests with at least two for each cognitive domain.

The Mini‐Mental Status Examination (MMSE), the Frontal Assessment Battery (FAB) and the Clock Drawing Test (CDT) were used as screening tests to evaluate global cognitive efficiency (MMSE), frontal lobe functions (FAB), and executive functions and visuospatial skills (CDT) [20, 21, 22].

For the memory domain it was administered the Rey Auditory Verbal Learning test (RAVLT) and the Rey–Osterrieth complex figure (ROCF) [23, 24]. Further tests for the executive functions included the Digit Span Backward (DS‐B), Verbal Fluency Test (VFT) semantic and phonemic, Trail Making Test version B (TMT‐B), and the Stroop Color‐Word Test (SCWT) [25, 26, 27]. The basic attention functions were assessed by the Digit Span Forward (DS‐F), the Trail Making Test version A (TMT‐A) [25, 26]. For the visuospatial domain, beside the CDT, it was administered the Italian Figure copy test, and the “Incomplete Letters” subtest of the Visual Object and Space Perception (VOSP) [28, 29]. The language domain assessment included the short version of Boston Naming Test (BNT‐15 items) [30]. Details of the neuropsychological tests and cognitive domains are summarized in Table S1.

### 2.3 Sample Collection and Storage

For serum collection, venous blood was drawn by venipuncture from all patients, then collected in serum separator tubes and allowed to clot for 30 min at room temperature. After centrifugation at 1000*g* (relative centrifugation force) for 20 min, serum samples were aliquoted into polypropylene tubes and stored at −80°C until analysis. For CSF sampling, all patients underwent lumbar puncture according to standard procedures. CSF samples were centrifuged at 2000*g* for 10 min at room temperature, then aliquoted and stored at −80°C.

### 2.4 Serum and CSF Irisin Assay

Serum and CSF irisin levels were determined by the competitive enzyme‐linked immunosorbent assay (ELISA) kit (EK‐067‐29, Phoenix Pharmaceuticals, Burlingame, CA, USA) according to the manufacturer's instructions. The assay has a sensitivity of 1.29 ng/mL with a detection range of 0.1–1000 ng/mL, and interassay and intra‐assay variation of < 15% and < 10%, respectively. Standard dilutions, positive controls, and patient samples were analyzed in duplicate using a plate reader (Eon, BioTek, Winooski, Vermont) with absorbance at 450 nm. Results were reported in nanograms per milliliter.

### 2.5 Statistical Analysis

Statistical analyses were performed using GraphPad Prism software version 9.5.0 (GraphPad Software, San Diego, CA) and SPSS version 22.0 (IBM, Armonk, NY). Distribution of the data was determined by the Shapiro–Wilk test and differences among groups were calculated using nonparametric Kruskal–Wallis test and Dunn multiple comparison test, and analysis of variance (ANOVA) with post hoc Tukey test. Correlations for pooled group of patients were performed using Spearman correlation coefficient test and partial correlation coefficient test adjusted for age and sex. Differences between groups and correlations were considered significant when *p* < 0.05.

## 3 Results

### 3.1 Demographic and Clinical Participant Characterization

As shown in Table S2, the present study cohort comprised 82 AD, 44 MCI, and 20 SMC patients equally balanced with respect to sex (*p* = 0.367). The mean age of patients with AD was > 65 years and significantly higher than SMC (*p* = 0.006). A higher education degree was observed in SMC compared to AD (*p* = 0.026). In addition, AD patients had significantly higher mean CDR and CDR‐SOB scores than MCI and SMC patients (*p* < 0.0001 for both).

### 3.2 Performance on Neuropsychological Examination

The participants of this study underwent an extensive neuropsychological battery. As expected, we found significant differences among SMC, MCI, and AD patients. In particular, as shown in Table S3, across all cognitive domains, patients with AD performed worse than those with MCI or SMC.

### 3.3 Serum Irisin Levels in AD, MCI, and SMC

We found a significant reduction of CSF and serum irisin levels in AD patients compared to SMC (5.65 ± 0.77 vs. 6.59 ± 1.34; *p* = 0.004). A trend toward a decrease was observed in AD compared to MCI patients (5.65 ± 0.77 vs. 6.24 ± 1.27; *p* = 0.074). No difference was noticed between patients with MCI and SMC (*p* = 0.433). Table S4 summarizes the results of this analysis.

### 3.4 Correlation Between Irisin and Cognition

**FIGURE 1:**
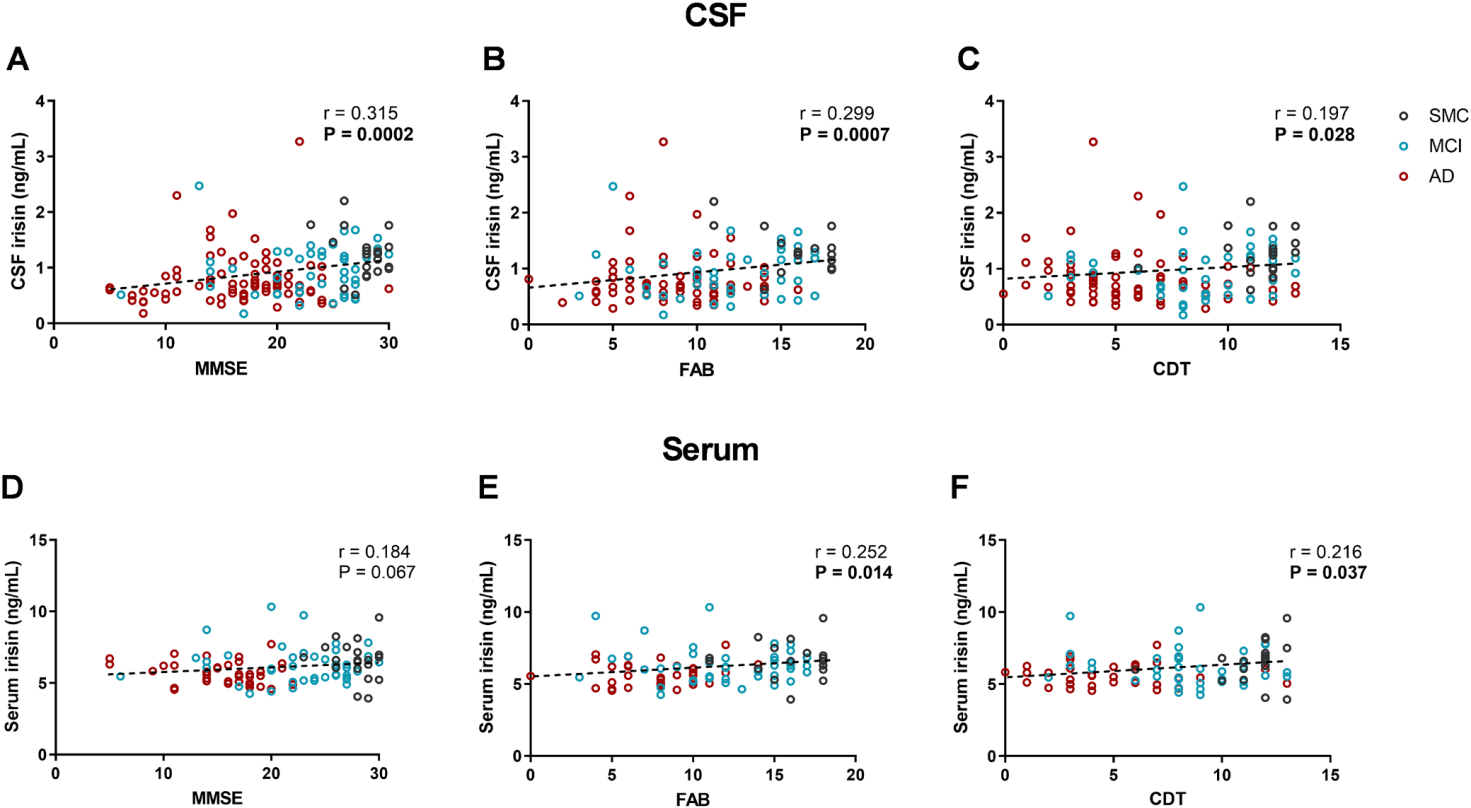
Correlations between CSF (A–C) or serum (D–F) irisin levels and screening test scores. Dotted lines represent Spearman linear regressions (*r* and *p* values as indicated). Bold values highlight statistically significant correlations. AD, Alzheimer's dementia; CDT, Clock Drawing Test; CSF, cerebrospinal fluid; FAB, Frontal Assessment Battery; MCI, mild cognitive impairment; MMSE, Mini‐Mental Status Examination; SMC, subjective memory complaints.

We observed significant positive correlations between CSF irisin levels and MMSE (*p* = 0.0002; *r* = 0.315), FAB (*p* = 0.0007), and CDT scores (*p* = 0.028; *r* = 0.197) (Figure 1A–C). For serum irisin levels, we found positive correlations with FAB (*p* = 0.014; *r* = 0.252), and CDT score (*p* = 0.037; *r* = 0.216), and a positive trend near statistical significance with MMSE (*p* = 0.067; *r* = 0.184) (Figure 1D–F).

**FIGURE 2:**
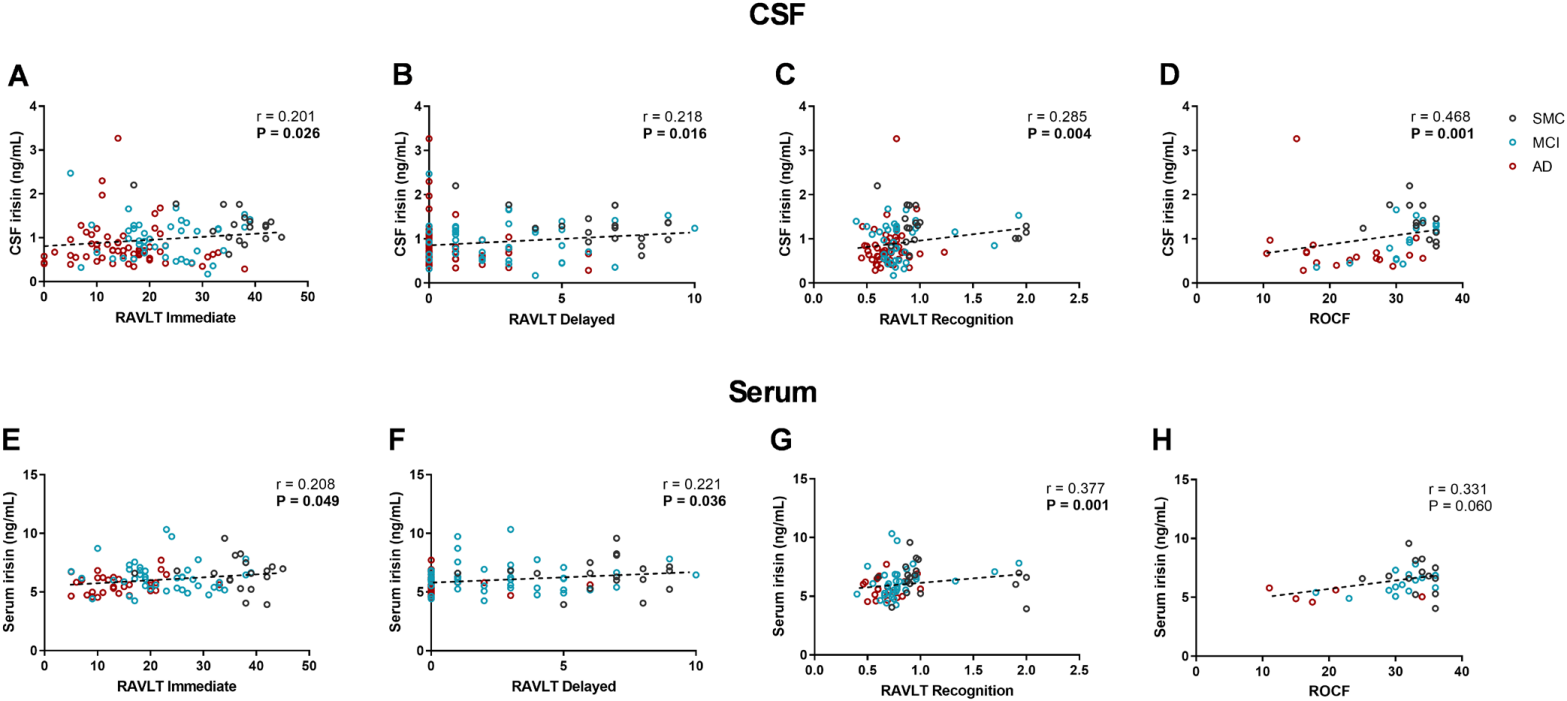
Correlations between CSF (A–D) or serum (E–H) irisin levels and memory test scores. Dotted lines represent Spearman linear regressions (*r* and *p* values as indicated). Bold values highlight statistically significant correlations. AD, Alzheimer's dementia; CSF, cerebrospinal fluid; MCI, mild cognitive impairment; RAVLT, Rey Auditory Verbal Learning Test; ROCF, Rey–Osterrieth Complex Figure; SMC, subjective memory complaints.

We noted positive correlations between CSF irisin levels and RAVLT immediate (*p* = 0.026; *r* = 0.201), delayed (*p* = 0.016; *r* = 0.218), and recognition score (*p* = 0.004; *r* = 0.285) (Figure 2A–C). Similarly to CSF, we found positive correlations between serum irisin levels and RAVLT immediate (*p* = 0.049; *r* = 0.208), delayed (*p* = 0.036; *r* = 0.221), and recognition score (*p* = 0.001; *r* = 0.377) (Figure 2E–G). For ROCF score, we observed a positive correlation with CSF irisin (*p* = 0.001; *r* = 0.468), and a positive trend near statistical significance for serum irisin (*p* = 0.060; *r* = 0.331) (Figure 2D,H).

**FIGURE 3:**
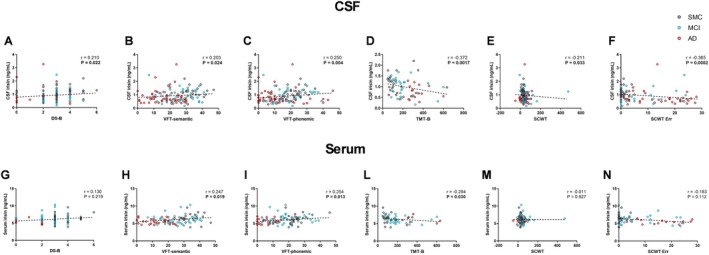
Correlations between CSF (A–F) or serum (G–N) irisin levels and executive function test scores. Dotted lines represent Spearman linear regressions (*r* and *p* values as indicated). Bold values highlight statistically significant correlations. AD, Alzheimer's dementia; CSF, cerebrospinal fluid; DS‐B, Digit Span Backward test; MCI, mild cognitive impairment; SCWT, Stroop Color and Word Test; SMC, subjective memory complaints; TMT‐B, Trail Making Test Version B; VFT, Verbal Fluency Test.

For CSF irisin, we found significant positive correlations with DS‐B (*p* = 0.022; *r* = 0.210), VFT semantic (*p* = 0.024; *r* = 0.203) and phonemic scores (*p* = 0.004; *r* = 0.250), and significant negative correlations with TMT‐B (*p* = 0.0017; *r* = −0.372), SCWT score (*p* = 0.033; *r* = −0.211), and SCWT errors (*p* = 0.0002; *r* = −0.365) (Figure 3A–F). For serum irisin, we observed a significant positive correlation with both VFT semantic (*p* = 0.019; *r* = 0.247) and phonemic scores (*p* = 0.013; *r* = 0.254), and a negative correlation with TMT‐B (*p* = 0.030; *r* = −0.294). No correlations were found with DS‐B (*p* = 0.219; *r* = 0.130), SCWT score (*p* = 0.927; *r* = −0.011), and SCWT errors (*p* = 0.112; *r* = −0.183) (Figure 3G–N).

**FIGURE 4:**
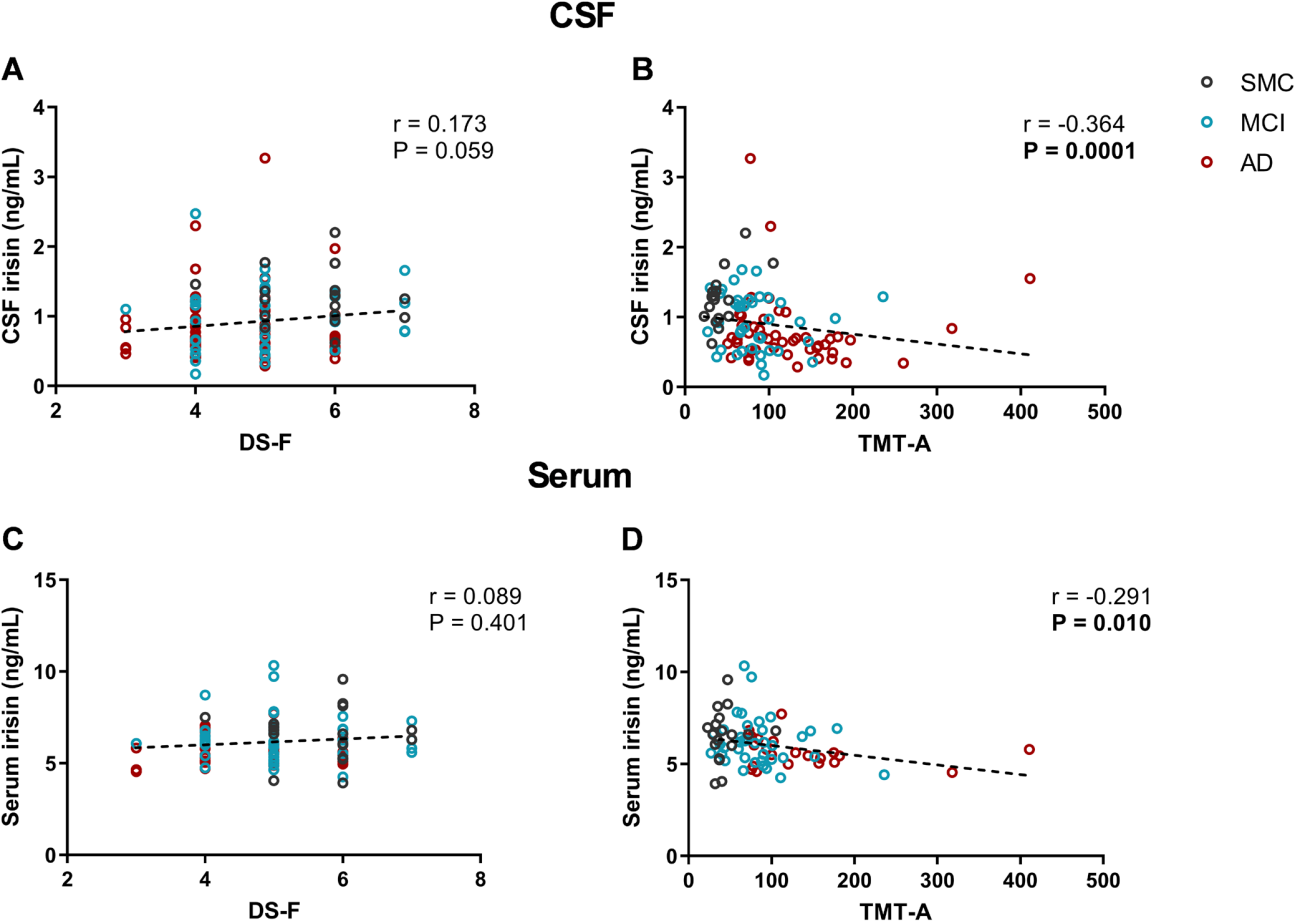
Correlations between CSF (A–B) or serum (C–D) irisin levels and attention test scores. Dotted lines represent Spearman linear regressions (*r* and *p* values as indicated). Bold values highlight statistically significant correlations. AD, Alzheimer's dementia; CSF, cerebrospinal fluid; DS‐F, Digit Span Forward; MCI, mild cognitive impairment; SMC, subjective memory complaints; TMT‐A, Trail Making Test version A.

We observed that TMT‐A score was negatively correlated with both CSF (*p* = 0.0001; *r* = −0.364), and serum irisin (*p* = 0.010; *r* = −0.291) (Figure 4B,D). For DS‐F score a positive trend was observed with CSF irisin (*p* = 0.059; *r* = 0.173), while there was no correlation with serum irisin levels (*p* = 0.401; *r* = 0.089) (Figure 4A,C).

**FIGURE 5:**
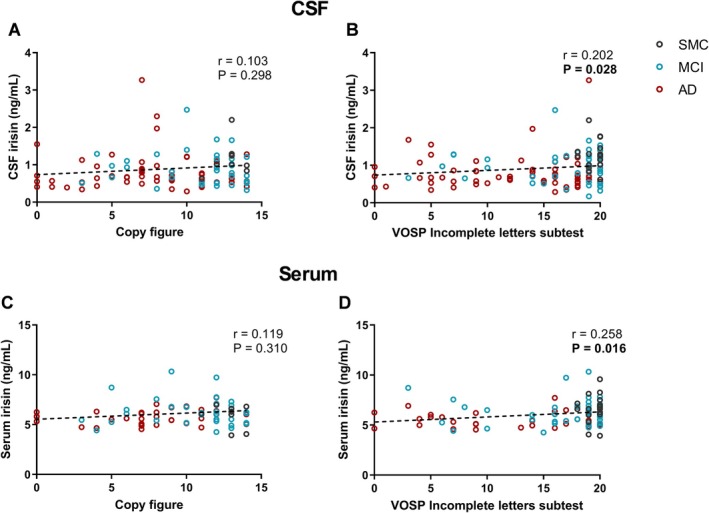
Correlations between CSF (A–B) or serum (C–D) irisin levels and visuospatial abilities's test scores. Dotted lines represent Spearman linear regressions (*r* and *p* values as indicated). Bold values highlight statistically significant correlations. AD, Alzheimer's dementia; CSF, cerebrospinal fluid; MCI, mild cognitive impairment; SMC, subjective memory complaints; VOSP, Visual Object and Space Perception.

For VOSP, we found significant positive correlations with both CSF irisin (*p* = 0.028; *r* = 0.202), and serum irisin (*p* = 0.016; *r* = 0.258) (Figure 5B,D). Figure copy score did not correlate with either CSF (*p* = 0.298; *r* = 0.103) or serum irisin levels (*p* = 0.310; *r* = 0.119) (Figure 5A,C).

**FIGURE 6:**
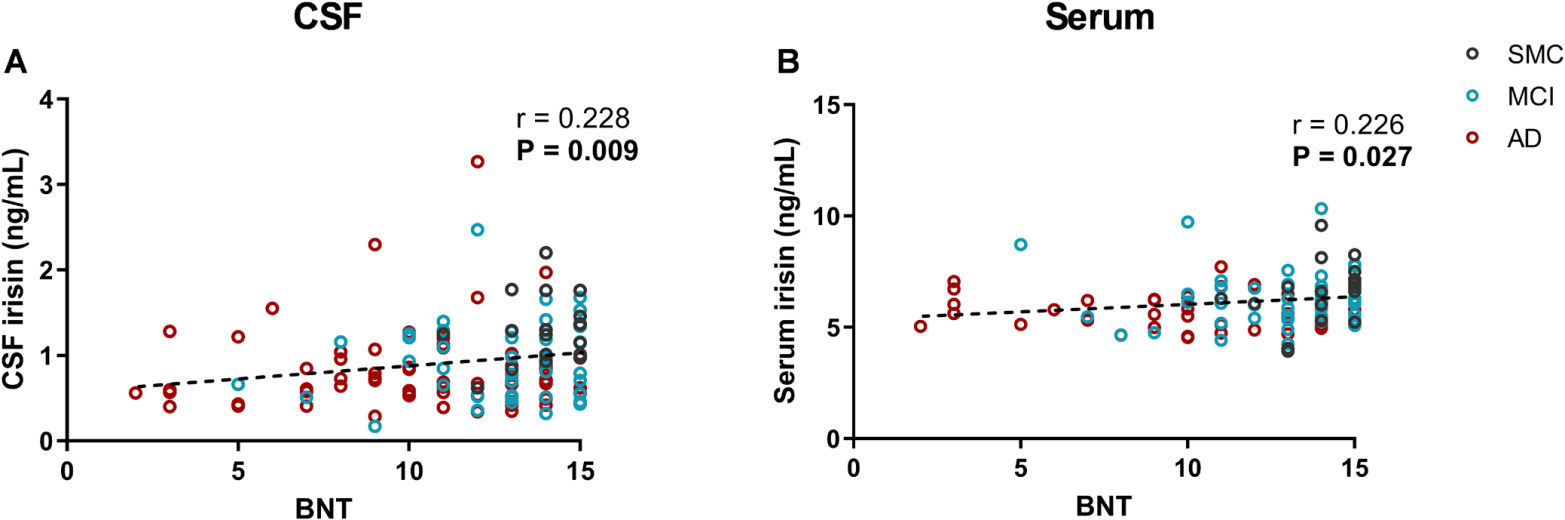
Correlations between CSF (A) or serum (B) irisin levels and language test scores. Dotted lines represent Spearman linear regressions (*r* and *p* values as indicated). Bold values highlight statistically significant correlations. AD, Alzheimer's dementia; BNT, Boston Naming Test; CSF, cerebrospinal fluid; MCI, mild cognitive impairment; SMC, subjective memory complaints.

Finally, for BNT we found a positive correlation with both CSF (*p* = 0.009; *r* = 0.228) and serum irisin levels (*p* = 0.027; *r* = 0.226) (Figure 6A,B).

Table 1 summarizes the results of these correlations.

**TABLE 1:** Correlations of CSF and serum irisin levels with cognitive scores.

	CSF irisin		Serum irisin	
	*r*	*p*	*r*	*p*
Screening tests				
MMSE	0.315	**0.0002**	0.184	0.067
FAB	0.299	**0.0007**	0.252	**0.014**
CDT	0.197	**0.028**	0.216	**0.037**
Memory				
RAVLT Immediate	0.201	**0.026**	0.208	**0.049**
RAVLT Delayed	0.218	**0.016**	0.221	**0.036**
RAVLT Recognition	0.285	**0.004**	0.377	**0.001**
ROCF	0.468	**0.001**	0.331	0.060
Executive functions				
DS‐B	0.210	**0.022**	0.130	0.219
VFT‐semantic	0.203	**0.024**	0.247	**0.019**
VFT‐phonemic	0.250	**0.004**	0.254	**0.013**
TMT‐B	−0.372	**0.0017**	−0.294	**0.030**
SCWT	−0.211	**0.033**	−0.011	0.927
SCWT Err	−0.365	**0.0002**	−0.183	0.112
Attention				
DS‐F	0.173	0.059	0.089	0.401
TMT‐A	−0.364	**0.0001**	−0.291	**0.010**
Visuospatial abilities				
Copy figure	0.103	0.298	0.119	0.310
VOSP Incomplete letters subtest	0.202	**0.028**	0.258	**0.016**
Language				
BNT	0.228	**0.009**	0.226	**0.027**

*Note:* Bold values highlight statistically significant correlations (Spearman correlation coefficient test; *r*, Spearman's correlation coefficient).

Abbreviations: BNT, Boston Naming Test; CDT, Clock Drawing Test; DS‐B, Digit Span Backward test; DS‐F, Digit Span Forward; FAB, Frontal Assessment Battery; MMSE, Mini‐Mental Status Examination; RAVLT, Rey Auditory Verbal Learning Test; ROCF, Rey–Osterrieth Complex Figure; SCWT, Stroop Color and Word Test; TMT‐A, Trail Making Test version A; TMT‐B, Trail Making Test Version B; VFT‐phonemic, Verbal Fluency Test‐phonemic; VFT‐semantic, Verbal Fluency Test‐semantic; VOSP, Visual Object and Space Perception.

### 3.5 Partial Correlations

All the correlations of CSF and serum irisin levels with global cognitive efficiency and cognitive domain scores were adjusted for age and sex as covariates. Table 2 summarizes the results of partial correlation analysis. For the correlations with CSF irisin levels, we observed that the positive correlations with MMSE (*p* = 0.005; *r* = 0.241), FAB (*p* = 0.038; *r* = 0.187), and BNT (*p* = 0.027; *r* = 0.197) scores and the negative ones with TMT‐B (*p* = 0.032; *r* = −0.262) score and SCWT errors (*p* = 0.036; *r* = −0.210) remained significant when corrected for age and sex.

**TABLE 2:** Partial correlations of CSF and serum irisin levels with cognitive scores.

Correlation	CSF irisin		Serum irisin	
	Age, Sex		Age, Sex	
	*r*	*p*	*r*	*p*
Screening tests				
MMSE	0.241	**0.005**	0.179	0.078
FAB	0.187	**0.038**	0.191	0.068
CDT	0.132	0.147	0.161	0.126
Memory				
RAVLT Immediate	0.152	0.096	0.148	0.170
RAVLT Delayed	0.164	0.072	0.157	0.145
RAVLT Recognition	0.188	0.060	0.163	0.165
ROCF	0.289	0.057	0.407	**0.023**
Executive functions				
DS‐B	0.126	0.177	0.122	0.253
VFT‐semantic	0.152	0.097	0.234	**0.027**
VFT‐phonemic	0.142	0.112	0.235	**0.023**
TMT‐B	−0.262	**0.032**	−0.217	0.118
SCWT	−0.045	0.659	0.024	0.839
SCWT Err	−0.210	**0.036**	−0.187	0.108
Attention				
DS‐F	0.119	0.199	0.117	0.271
TMT‐A	−0.178	0.073	−0.260	**0.023**
Visuospatial abilities				
Copy figure	0.071	0.477	0.114	0.336
VOSP Incomplete letters subtest	0.168	0.072	0.164	0.135
Language				
BNT	0.197	**0.027**	0.115	0.268

*Note:* Bold values highlight statistically significant correlations.

Abbreviations: BNT, Boston Naming Test; CDT, Clock Drawing Test; DS‐B, Digit Span Backward test; DS‐F, Digit Span Forward; FAB, Frontal Assessment Battery; MMSE, Mini‐Mental Status Examination; RAVLT, Rey Auditory Verbal Learning Test; ROCF, Rey–Osterrieth Complex Figure; SCWT, Stroop Color and Word Test; TMT‐A, Trail Making Test version A; TMT‐B, Trail Making Test Version B; VFT‐phonemic, Verbal Fluency Test‐phonemic; VFT‐semantic, Verbal Fluency Test‐semantic; VOSP, Visual Object and Space Perception.

For the correlations with serum irisin levels, we found that the positive correlation with ROCF (*p* = 0.023; *r* = 0.407), VFT‐semantic (*p* = 0.027; *r* = 0.234) and VFT‐phonemic (*p* = 0.023; *r* = 0.235) scores and the negative correlation with TMT‐A (*p* = 0.023; *r* = −0.260) score remained significant when corrected for age and sex. Trends of significance were noted for the positive correlations with MMSE (*p* = 0.078; *r* = 0.179) and FAB (*p* = 0.068; *r* = 0.191) scores.

## 4 Discussion

In the present study, we evaluated the relationship between CSF or serum irisin levels and global and domain‐specific cognitive performance in the continuum of AD. Our results showed that CSF irisin levels were significantly correlated with all the cognitive domains, mainly with the scores of global cognitive efficiency (MMSE), memory (ROCF test), executive functions (TMT‐B and SCWT), and attention (TMT‐A). For serum irisin levels, the correlations were, for the most part, similar to those observed for CSF irisin except for the MMSE, ROCF and DS‐B test, and SCWT in which trends to significance were noted.

Favorable lifestyle habits, including regular PE, have positive effects in the delay of brain aging and are promoted as preventative strategies to counteract the physiological age‐related memory loss and neurodegeneration in older healthy subjects [31]. Indeed, multidomain interventions, including PE, which address multiple risk factors associated with dementia, have been shown to be effective in preventing and/or delaying cognitive decline [32]. Remarkable evidence suggests that regular PE positively impacts memory performance in older adults [33]. Aerobic PE has been the most widely employed approach to examine how PE can mitigate the detrimental effects of aging on cognitive performance; however, in the last years, the impact of resistance exercise on cognitive functions has gained importance [31].

Noteworthy, the benefits of PE on brain health are also evident in patients with MCI and AD who, despite the onset of a cognitive decline, displayed an improvement in their cognitive functions when engaged in aerobic or resistance exercise programs [31]. In particular, it has been demonstrated that PE mainly improved executive functions, memory, and attention in both MCI and AD patients [31].

Among the theories proposed to explain the positive impact of PE on brain functions, an increasing number of studies have focused on skeletal muscle‐brain crosstalk, highlighting the neuroprotective effects of soluble factors, that is, myokines, released in the circulation by muscles in response to PE [34]. In this context, the pathway involving the myokine irisin and its precursor FNDC5 has received so much interest that numerous animal experiments and human studies have contributed to proving the neuroprotective role of FNDC5/irisin in the central nervous system through different molecular mechanisms [35]. Further studies on elderly patients have shown that the genetic variants of FNDC5 are linked to reduced brain glucose metabolism, suggesting that FNDC5 might be involved in the control of metabolism in brain areas susceptible to the pathophysiology of AD [36].

Of interest, several reports suggested the relevance of FNDC5/irisin in protecting against disorders associated with cognitive decline, such as AD and depression [37, 38]. Interestingly, recent evidence showed molecular similarities involving the FNDC5/irisin system and other factors, such as neurotrophins, in elderly individuals affected by dementia or depression [38]. Hippocampal FNDC5/irisin expression was significantly reduced in different AD mouse models that exhibited cognitive deficits [10, 11]. However, cognitive impairments were rescued by directly increasing FNDC5/irisin levels in the brain [11] or boosting irisin concentration in the blood via adenoviral FNDC5 overexpression in the liver [10].

Despite the findings in AD models, the link between irisin and cognitive functions has been poorly investigated in cohorts of AD patients.

To investigate on this topic, in the present paper we correlated CSF or serum irisin levels with cognitive domains in the continuum of AD; thus, we enrolled patients with SMC, MCI, and AD dementia biologically classified according to the 2018 ATN scheme of the NIA‐AA [15].

Of note, we found significant correlations between CSF irisin and all the cognitive domains; indeed, higher CSF irisin levels were associated with better cognitive performances.

For global cognition efficiency, our results corroborated the positive correlation between CSF irisin and MMSE previously demonstrated by Lourenco et al. in a cohort of AD patients and nondemented controls [14].

The positive correlations between CSF irisin and memory domain reinforced the key role of the FNDC5/irisin pathway in regulating the memory process [35]. For instance, Lourenco et al. [11] demonstrated that the knockdown of brain FNDC5/irisin impaired memory in a novel object recognition (NOR) task and synaptic plasticity in wild‐type (WT) mice. Recent investigations in global *Fndc5* knock‐out (F5KO) mice evidenced less PE‐induced improvements in spatial learning and memory in young and aged F5KO mice compared to the WT [10].

As novelty, the correlations between CSF irisin levels and unexplored cognitive functions such as executive functions, attention, visuospatial abilities, and language showed significant results that suggested the positive impact of FNDC5/irisin on different cognitive domains, besides memory.

To assess whether also circulating irisin reflected the cognitive decline in the continuum of AD, we first compared serum irisin concentration in the study cohort of patients. Of note, like CSF irisin [12], here we found reduced serum irisin levels in AD and MCI patients compared to SMC.

The results of the correlation analysis for serum irisin overlapped for the most part with those observed for the CSF, evidencing the potential of serum irisin as a cognitive biomarker to track the progression of AD. In accordance with our data, positive associations of serum irisin levels with global cognition and episodic memory have been demonstrated in a population of older adults at risk of AD [39]. Similarly, Tsai and Pai proposed serum irisin as an influencing factor of the visuospatial working memory in obese individuals at genetic risk for AD [40]. Our data are in contrast with those of a recent study that reported the loss of association between plasma irisin levels and cognition in AD [13]. However, this divergence was possibly due to a different statistical approach as we evaluated all the correlations in the continuum of AD by merging the groups of patients, while the other study considered non‐AD, MCI, and AD participants separately [13].

Demographic factors, such as age and sex, can impact on cognitive domains in AD [41]. When we statistically accounted for these variables in our analysis, we noticed that several correlations maintained their significance even after adjustment for age and sex.

Our study has some limitations that should be mentioned. First, irisin levels could be affected by different factors, including PE [42] and drug treatments for concomitant diseases [43, 44]. In the present study cohort, we did not collect patients self‐reported information regarding the level of their physical activity; however, we did not find differences among patients in a proxy indicator of PE, that is, body mass index, nor correlations with CSF or serum irisin levels (data not shown).

Tandem mass spectrometry evidenced significant individual differences in CSF irisin levels in elderly, possibly due to various diseases and concomitant pharmacological therapies [44]. Thus, further studies including PE and drug treatment information will be necessary to better address the clinical significance of irisin as biomarker for cognitive impairment in AD.

Second, our results evidenced irisin impact on various cognitive domains, however, we did not examine the molecular mechanism that explained our observational data. At present, FNDC5/irisin system has been mainly investigated in the hippocampus, involved in memory and learning [9, 11]. In future analysis, it would be desirable to examine how FNDC5/irisin system acts in other cerebral areas as well. Finally, we primarily used correlation analysis that, together with the cross‐sectional nature of this study, did not allow to infer a clear causality in the association between irisin and cognition. Longitudinal studies need to be performed after this preliminary explorative analysis.

The present study extended the findings of our previously published article that showed the association between irisin levels and AD biomarkers, evidencing the role for FNDC5/irisin system in protecting against cognitive decline for multiple cognitive domains. The strength and novelty of the present work is the use of a patient cohort stratified based on their biomarker profiles for Aβ, tau, and neurodegeneration.

In conclusion, our results encourage further investigations suggesting FNDC5/irisin pathway as a potential target for therapeutic strategies to counteract cognitive impairment in AD patients. Furthermore, serum irisin with its feasibility and accessibility could be a candidate biological marker for cognitive decline in AD patients.

## Author Contributions

M.G., G.L., and S.C. designed the study. P.P., M.D., D.U., C.Z., D.V., M.T.D., F.B., R.Z., A.O., and G.C. conducted the research. P.P., M.D., D.U., C.Z., D.V., M.T.D., F.B., R.Z., A.O., G.C., S.C., and M.G. performed data analysis. M.D., P.P., C.Z., M.T.D., D.U., F.B., R.Z., A.O., G.C., S.C., G.L., and M.G. discussed the results. P.P., M.D., D.U., C.Z., D.V., M.T.D., S.C., M.G., and G.L. wrote the manuscript.

## Conflicts of Interest

The authors declare no conflicts of interest.

## Supporting information


**Table S1.** Cognitive domains and neuropsychological tests administered to the study participants.

**Table S2.** Participant demographic and clinical data.

**Table S3.** Cognitive scores in SMC, MCI, and AD patients.

**Table S4.** CSF and serum irisin levels in SMC, MCI, and AD dementia patients.

## Data Availability

The data that support the findings of this study are available from the corresponding author upon request.
