# Supplementary material for: Correlation Between Irisin and Cognitive Functions in Alzheimer Dementia
Source: Ann Clin Transl Neurol. 2025 Jun 25;12(9):1743–52. doi: 10.1002/acn3.70117 (PMC12455884; doi:10.1002/acn3.70117)
Supplement: Supplementary file 1 — Table S1. Cognitive domains and neuropsychological tests administered to the study participants. [file ACN3-12-1743-s002.docx]

**SUPPLEMENTARY TABLE S1.** Cognitive domains and neuropsychological tests administered to the study participants.

|  | **Neuropsychological Tests** |
| --- | --- |
| **Screening tests** |  |
|  | Mini-Mental Status Examination (MMSE)  Frontal Assessment Battery (FAB)  Clock Drawing Test (CDT) |
| **Cognitive domains** |  |
| Memory | Rey Auditory Verbal Learning Test (RAVLT)   - Immediate - Delayed - Recognition   Rey–Osterrieth Complex Figure (ROCF) |
| Executive functions | Digit Span- Backward (DS-B)  Verbal Fluency Test (VFT) - Phonemic  Verbal Fluency Test (VFT) - Semantic  Trail Making Test version B (TMT-B)  Stroop Color and Word Test (SCWT)   - Stroop Color and Word Test (SCWT) - Stroop Color and Word Test (SCWT) Error |
| Attention | Digit Span - Forward (DS-F)  Trail Making Test version A (TMT-A) |
| Visuospatial abilities | Copy figure  Visual Object and Space Perception (VOSP) incomplete letters subtest |
| Language | Boston Naming Test (BNT) – short version |
