# Supplementary material for: Correlation Between Irisin and Cognitive Functions in Alzheimer Dementia
Source: Ann Clin Transl Neurol. 2025 Jun 25;12(9):1743–52. doi: 10.1002/acn3.70117 (PMC12455884; doi:10.1002/acn3.70117)
Supplement: Supplementary file 2 — Table S2. Participant demographic and clinical data. [file ACN3-12-1743-s001.docx]

**SUPPLEMENTARY TABLE S2**. Participant demographic and clinical data

| Patient characteristics | SMC | MCI | AD | *P* value | SMC vs MCI | SMC vs AD | MCI vs AD |
| --- | --- | --- | --- | --- | --- | --- | --- |
| Age, yr | 60.35 ± 10.97  59 (50-69.25) | 63.64 ± 10.74  64 (56.25-71) | 67.51 ± 7.81  68 (62-73.25) | **0.003** | 0.387 | **0.006** | 0.067 |
| Sex, n (%) |  |  |  |  |  |  |  |
| Women | 9 (45) | 25 (57) | 51 (62) |  |  |  |  |
| Men | 11 (55) | 19 (43) | 31 (38) | 0.367 vs. women |  |  |  |
| Education, yr | 11.05 ± 3.71  11.50 (8-13.75) | 9.16 ± 3.98  8 (5.75-13) | 8.61 ± 4.72  8 (5-13) | **0.029** | 0.283 | **0.026** | 0.828 |
| CDR total | 0.03 ± 0.11  0 (0-0) | 0.5 ± 0.11  0.5 (0.5-0.5) | 1.22 ± 0.76  1 (1-1) | **<0.0001** | **0.001** | **<0.0001** | **<0.0001** |
| CDR-SOB | 0.33 ± 0.49  0 (0-0.5) | 2.42 ± 1.49  2.5 (1.5-3.5) | 5.92 ± 4.05  4.5 (3.5-7.5) | **<0.0001** | **0.007** | **<0.0001** | **<0.0001** |

Notes: Values are presented as mean ± SD; Median (Interquartile range Q1-Q3). Bold values highlight statistically significant differences among patient groups (Pearson’s chi squared test, *P*<0.05, for sex; ANOVA-Tukey test and Kruskal-Wallis-Dunn’s test, *P* <0.05, for continuous data).

Abbreviations: SMC, subjective memory complaints; MCI, mild cognitive impairment; AD, Alzheimer’s dementia; CDR, Clinical Dementia Rating; CDR-SOB, CDR scale Sum of Boxes; SD, standard deviation; Q1, lower quartile; Q3, upper quartile.
