# Supplementary material for: Correlation Between Irisin and Cognitive Functions in Alzheimer Dementia
Source: Ann Clin Transl Neurol. 2025 Jun 25;12(9):1743–52. doi: 10.1002/acn3.70117 (PMC12455884; doi:10.1002/acn3.70117)
Supplement: Supplementary file 4 — Table S4. CSF and serum irisin levels in SMC, MCI, and AD dementia patients. [file ACN3-12-1743-s003.docx]

**SUPPLEMENTARY TABLE S4.** CSF and serum irisin levels in SMC, MCI, and AD dementia patients.

| **Biological fluids of participant** | **SMC** | **MCI** | **AD** | ***P* value** |
| --- | --- | --- | --- | --- |
| CSF irisin, pg/ml (10) | 1.23 ± 0.42;  1.25 (0.94-1.44) | 0.95 ± 0.45;  0.93 (0.53-1.25) | 0.80 ± 0.47;  0.69 (0.53- 0.91) | **0.046** MCI vs. SMC  **< 0.0001** AD vs. SMC  0.079 AD vs. MCI |
| Serum irisin, pg/ml* | 6.59 ± 1.34;  6.61 (6.02-7.41) | 6.24 ± 1.27;  6.11 (5.36-6.82) | 5.65 ± 0.77;  5.59 (4.98-6.21) | 0.433 MCI vs. SMC  **0.004** AD vs. SMC  0.074 AD vs. MCI |

Notes: Data are presented as mean ± SD; Median (Interquartile range Q1-Q3). Bold values highlight statistically significant differences (Kruskal-Wallis-Dunn’s test, *P* < 0.05).

Abbreviations: SMC, subjective memory complaints; MCI, mild cognitive impairment; AD, Alzheimer’s dementia; CSF, cerebrospinal fluid; SD, standard deviation.

* Data not available for all participants (n = 20 for SMC, n = 44 for MCI, and n = 42 for AD patients).
